# The progress test of medicine: the Dutch experience

**DOI:** 10.1007/s40037-015-0237-1

**Published:** 2016-01-11

**Authors:** René A. Tio, Bert Schutte, Ariadne A. Meiboom, Janke Greidanus, Eline A. Dubois, Andre J. A. Bremers, the Dutch Working Group of the Interuniversity Progress Test of Medicine

**Affiliations:** 1 Department of Cardiology, University Medical Center Groningen, University of Groningen, 9700 PO Box 30.001, RB Groningen, The Netherlands; 2 Maastricht University, Maastricht, The Netherlands; 3 VU University Medical Center, Amsterdam, The Netherlands; 4 University Medical Center Groningen, University of Groningen, Groningen, The Netherlands; 5 Leiden University Medical Center, Leiden, The Netherlands; 6 Department of Surgery, Radboud University Medical Center, Nijmegen, The Netherlands

**Keywords:** Benchmarking, Formative assessment, Progress test, Summative assessment

## Abstract

Progress testing in the Netherlands has a long history. It was first introduced at one medical school which had a problem-based learning (PBL) curriculum from the start. Later, other schools with and without PBL curricula joined. At present, approximately 10,000 students sit a test every three months. The annual progress exam is not a single test. It consists of a series of 4 tests per annum which are summative in the end. The current situation with emphasis on the formative and summative aspects will be discussed. The reader will get insight into the way progress testing can be used as feedback for students and schools.

## Introduction

A true problem-based learning (PBL) curriculum ‘aims at acquisition and structuring of knowledge …. in an active iterative and self-directed way’ [1]. Critics may question the validity of such a programme and argue that students taught in this way may develop deficiencies in their knowledge [2]. It is a challenge to develop an assessment programme fit for such a curriculum. Assessment of knowledge and even more so monitoring knowledge growth may be considered a requirement for external and internal validation of a PBL curriculum and also other curricula. In order to address this and to prove that knowledge acquisition is at the required level, progress testing was introduced in the 1970s in Missouri and Maastricht [3, 4]. The use of progress testing has increased ever since. Nowadays there is no continent (except for Antarctica) where progress testing is not used [5]. In this short overview we describe the present situation including the formative and summative aspects of progress testing in the Netherlands. Furthermore, its use for benchmarking will be discussed.

Many things have been changed since the first introduction of progress testing in the Netherlands. Initially, only one of the eight medical schools used it. Since the 1990s the number has increased rapidly and at present five schools are participating in the Dutch progress test and a sixth will start in the academic year 2015–2016. This means that more than 10,000 students sit the exam at the same time. In our collaboration we plan the dates well ahead taking into account local logistics and local and national holidays. The exam consists of 4 quarterly tests of 200 items each. These items are distributed according to a fixed two-dimensional matrix (Table 1). Using a test with 200 items 4 times a year has a high reliability for all the year cohorts. Cronbach’s alpha ranged from 0.898 to 0.943 with a mean of 0.92 during the period from 2005 to 2011. Furthermore, using such a high number of items per test also introduces adequate reliability for large subcategories of items within the test [6].

**Table 1:** Disciplines and categories of the Dutch progress test of medicine.

Disciplines	Categories
Anatomy	Respiratory system
Biochemistry	Blood & immune system
Surgery	Musculoskeletal system
Dermatology	Mental health care
Epidemiology	Reproductive system, pregnancy, childbirth & puerperium
Pharmacology	Cardiovascular system
Physiology	Hormones & metabolism, endocrine system
Obstetrics and gynaecology	Dermis & connective tissue
General practice	Personal and social aspects
Internal medicine	Digestive/gastrointestinal system, nutritional disorders
Paediatrics	Nervous system & senses
Ear nose throat	Kidneys & urinary system
Clinical genetics	Molecular & cellular aspects
Metamedical sciences	Epistemology, methodology & applied biostatistics
Neurology	Stages of life
Ophthalmology	Knowledge of skills
Pathology	Preventive medicine
Psychology and psychiatry	
Social medicine	

The blueprint of the test is two-dimensional

During the evolution of the test from one single institution to a multicentre test, results have continuously been evaluated and whenever possible improvements implemented. This is illustrated by the following example. In the beginning of the cooperation, Maastricht students scored better than those of the other participating schools. This was related to the fact that most questions originated from Maastricht at that time. This was a strong impulse for the other participating schools to increase item production and now all schools contribute equally to each test [7]. In this way none of the students benefit because the test has more familiar items or more items related to specific issues highlighted more in one and less in another curriculum. Nowadays no large differences between the participating schools are present. In order to maintain quality of test items all items have to fulfil strict criteria regarding item construction, and literature references. All items are first seen by a local review committee, if necessary rewritten, and then enter a national review process before they can be used in a test. After each test all students can send in commentary on items they think are not correct. These comments are first discussed in the local review committees. Subsequently, the final decision about questionable items is made in a national meeting.

A test which is conducted at different schools is a powerful instrument to compare curricula [8]. In our case the proportion of PBL in the different curricula varies from traditional (non-PBL), a hybrid between traditional and PBL to almost completely PBL. This gives the possibility to pursue the question whether students in a PBL school perform similarly to those in a non-PBL school. This was investigated in a previous paper. Although only two tests were taken into account, overall no systematic differences were found. However, in subcategories differences were present. Students from non-PBL schools scored higher on basic science items whereas students from a PBL school scored better on social science items [9]. In this way differences between schools and between cohorts can be monitored. Such data can be useful for comparing curricula and for evaluation of curriculum changes, students’ achievements and relationship between learning domains [10, 11].

Since the test is a test at the end level, it cannot be expected that undergraduate students know all the study material. Therefore, in case of progress testing the choice has to be made between forcing students to guess or giving them the opportunity to acknowledge that they do not know. Since we feel that it is important for students to learn that they cannot know everything we use the question mark option. This gives students the opportunity to acknowledge if they do not know the answer. Since the progress test uses this form of marking we could evaluate it in a real-life setting. For this purpose students were asked to indicate the option they thought the most correct when they did not know the answer. We observed that formula scoring yielded a lower percentage of correctly answered questions. This favours the assumption that partial knowledge can better be mobilized by forcing them to answer (guess) all questions [11]. Although psychometric analysis showed that formula scoring may be a disadvantage for students who are less inclined to guess, other educational considerations as mentioned above should also be valued. Furthermore, as far as reliability of a test is concerned, it has previously been shown that formula scoring tests may perform better than number right scoring tests, [12, 13] as well as worse [14].

For each test students receive a score Good/Pass/Fail. A relative standard setting is used, taking into account the mean and standard deviation of all year cohorts. The standards increase with the progress in their study. Each following test requires a higher score to get a pass. At the end of each year students receive an overall pass or fail for the exam based on the combination of the 4 tests. In this way the pass-fail decision of the progress test exam is never based on a single measurement but on a combination of 4. The overall criteria to pass the exam is that each year an adequate level of knowledge is acquired, which is reflected in sufficient ‘pass’ or ‘good’ scores. In case of one or more ‘fails’ this should be compensated for by sufficient ‘pass’ and ‘good’ scores. Since the test is conducted at 5 different schools, the greatest care is given to aligning the summative decisions. For this purpose a nationwide way of translating the results of the 4 formative tests into a summative decision (fail, pass or good) has been accepted. This resulted in a table in which all possible combinations (81) are included, each with their corresponding summative result. Although we agree upon this as national working group, the final decision lies with each local board of examiners. In order to prevent differences that may also influence the results, the tendency is that the general policy is taken over by all the local boards, which is the case for this table with all the combinations.

**Fig. 1:**
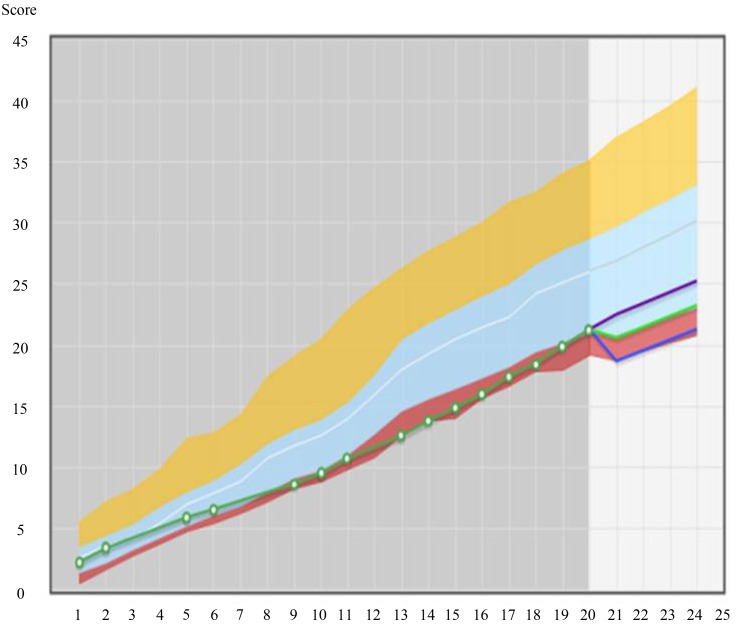
The PROgress test Feedback system (PROF). Longitudinal results of an individual student. The scores of an individual student after 20 test moments are shown. The *green line* represents the results of the student on the previous tests. The *red, blue* and *yellow* shaded areas represent the areas for fail, pass, and good scores. The *blue line* indicates the upper and lower limits of the likely future development in this student.

**Fig. 2:**
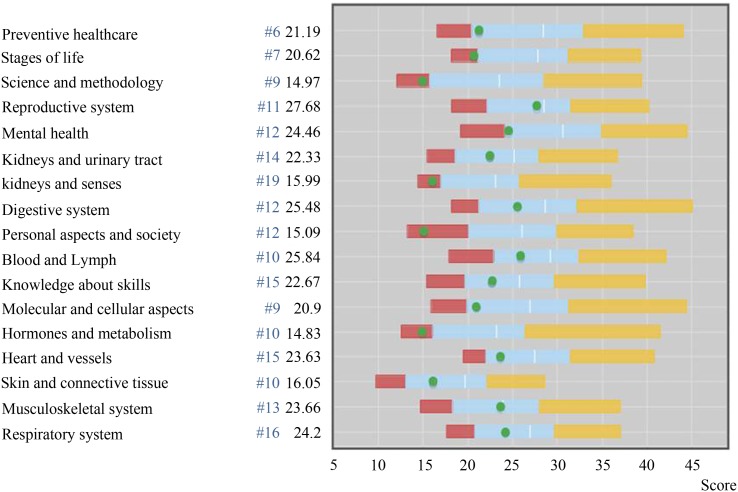
The PROgress test Feedback system (PROF). Scores of a student per category. The scores of a student on a test are shown per category. The *green dots* as well as the numbers without *#*, represent the actual scores. The *red, blue* and *yellow* shaded areas represent the areas for insufficient, sufficient, and good. The numbers with a *#* indicate the numbers of question per category.

The assumption that assessment drives learning is a widely accepted dogma in education [4, 14–17]. The items in each progress test are distributed according to a fixed two-dimensional matrix (Table 1). After each test students are allowed to take the test booklet with them and the answer key is published shortly after. In this way they can check their answers and identify their deficiencies. Since each of the quarterly tests has the same item distribution they can improve their score in certain subcategories in the following tests. In addition we constructed an online feedback system called PROgress test Feedback system ‘PROF’ (Fig. 1 and Fig. 2). This system allows students to gain understanding in their overall score (Fig. 1) as well as their scores per discipline or per category (Fig. 2) and to compare their own score with the average in their peer group, per test moment but also longitudinally [18]. In the context of this continuous and repeated testing and feedback, we have constructed a powerful tool to stimulate students to repair their deficiencies. A higher use of the PROF system was also associated with a higher knowledge growth (Donkers et al. submitted for publication) [19]. In this context it is important to mention that progress testing is also a valuable tool to use as a formative assessment monitoring knowledge growth [20].

Finally, it should be realized that a progress test is not the only assessment in a curriculum. It is part of the complete assessment programme which often includes block tests and assessment of skills and competencies by a wide variety of assessment tools. As such it can be used outside the framework of constructive alignment as it is an assessment in addition to all other assessments. It should be realized that it could be the most important (if not the only) knowledge assessment of a curriculum.

## Conclusion

The Dutch progress test is extraordinary for several reasons. It is a curriculum-independent test in which 5 medical schools cooperate in test production, as well as testing and scoring students. It combines formative and summative aspects of assessment. It is a curriculum-independent assessment at the end level of the medical curriculum. Finally, it is a rich source of information for students, researchers, schools and policymakers, for instance for comparing curricula and monitoring curricular changes.
